# An Approach to Sustainable Metrics Definition and Evaluation for Green Manufacturing in Material Removal Processes

**DOI:** 10.3390/ma13020373

**Published:** 2020-01-14

**Authors:** César Ayabaca, Carlos Vila

**Affiliations:** 1 Department of Mechanical Engineering and Materials, Universitat Politècnica de València, 46022 València, Spain; carvipas@upv.es; 2 Department of Mechanical Engineering, Faculty of Mechanical Engineering, Escuela Politécnica Nacional, Quito 170524, Ecuador

**Keywords:** green manufacturing, sustainability metrics, cleaner product life cycle, material removal processes

## Abstract

Material removal technologies should be thoroughly analyzed not only to optimize operations but also to minimize the different waste emissions and obtain cleaner production centers. The study of environmental sustainability in manufacturing processes, which is rapidly gaining importance, requires activity modeling with material and resource inputs and outputs and, most importantly, the definition of a balanced scorecard with suitable indicators for different levels, including the operational level. This paper proposes a metrics deployment approach for the different stages of the product life cycle, including a conceptual framework of high-level indicators and the definition of machining process indicators from different perspectives. This set of metrics enables methodological measurement and analysis and integrates the results into aggregated indicators that can be considered for continuous improvement strategies. This approach was validated by five case studies of experimental testing of the sustainability indicators in material removal operations. The results helped to confirm or modify the approach and to adjust the parameter definitions to optimize the initial sustainability objectives.

## 1. Introduction

The analysis of industrial manufacturing processes from the sustainability point of view started during the early 1980s in order to meet the sustainable development concept that arose in the 1970s as a result of a general worry about the global environment due to pollution and the consumption of energy and raw materials. In 1983, the United Nation (UN)’s World Commission on the Environment and Development, known as the Brundtland Commission, prepared a formal report entitled “Our Common Future”, in which the concept of sustainable development was defined as “development that meets the current needs of people without compromising the ability of future generations to meet theirs” [1]. Global movements and policies were generated in order to find common strategies that could be applied worldwide [2,3].

Subsequently, in the area of product manufacturing, many research projects were initiated to generate proposals for process improvement and the optimization of the consumption of resources and raw materials [4,5,6] as well as rules and regulations for waste management for cleaner production methods [7]. At the UN summit in 2015, the 17 goals for sustainable development for 2015 to 2030 were laid down, including goal number 9 (Industry, Innovation, and Infrastructure) which is focused on building resilient infrastructures, promoting inclusive and sustainable industrialization, and fostering innovation [8]. Among others, we could list the most important international initiatives that encourage a better understanding of sustainability:

(a) United Nations, Sustainable Development Goals.
(b) ISO 26000: 2010 Social Responsibility [9].
(c) United Nations Global Compact (Global Compact) [10].
(d) Guide for Organisation for Economic Co-operation and Development (OECD) multinationals. (Global Reporting Initiative) [11].

Since the end of the last century, researchers, technicians, managers, and environmentalists have recommended three economic, environmental, and social dimensions for evaluating economic, environmental, and social aspects, which are known as sustainability dimensions [12].

We must underline the contribution of Zackrisson et al. [13], who studied the relationship between performance measurement systems and sustainability. Although the research was done in Swedish manufacturing companies, they found that at the shop floor level, 90% of the indicators have at least an indirect relationship with one or more of the economic, environmental, or social dimensions, while 26% of the indicators are indirectly related to the environmental dimension. 

In materials and manufacturing engineering research topics, indicators are being proposed to measure the sustainability of industrial processes. Reich-Weiser et al. defined a general set of metrics for sustainable manufacturing [14], while Shuaib et al. [15] and Jayal et al. [16] designed a framework and a model for sustainable manufacturing, respectively. Singh et al. developed an expert system for the performance evaluation of small and medium enterprises [17]. With a greater focus on machining technologies, we find the work of Rajurkar et al., which explored how to ensure sustainability and optimize non-traditional machining processes [18].

Since machining technologies pollute and consume energy and raw materials, it is understandable that more research actions are needed. This work is part of the research into the design of green manufacturing activities within the product life cycle, focusing on machining technologies. This work will propose a framework and a list of indicators that will help to get data and information from manufacturing activities as part of the Life Cycle Assessment. The paper is structured into six sections. The Section 2 reviews the state of the art of green manufacturing applied to machining and presents our vision of a green manufacturing activities model. The Section 3 describes the framework for defining the metrics from the point of view of materials, parts, and processes during the product life cycle. The Section 4 describes the validation experiments, while the conclusions and future work are presented in the last two sections. 

## 2. State of the Art in Green Machining Operations

### 2.1. Analysis of Previous Works of Sustainability in Industrial Manufacturing Processes

The aim of this work was to carry out several experiments to calculate different types of sustainable indicators with different materials, processes, and machining centres. Many studies have been carried out on sustainable manufacturing and, in the literature, we can find many focused on specific disciplines, such as machining technologies. Peralta et al. [19] showed the trends emerging in the last 15 years in the sustainability of machining processes from the point of view of the triple bottom line and the three general dimensions: economic, ecological, and equity. Bhanot et al. [20] presented a statistically validated study that proposed a comprehensive sustainability framework for the manufacturing domain to strengthen the enablers and mitigate barriers based on the responses of researchers and industry professionals.

Eastwood et al. [21] developed a sustainable assessment methodology to both improve the accuracy of the existing approaches in identifying the sustainability impact of a product and to assist manufacturing decision makers using unit process modeling and life cycle inventory techniques. The proposed methodology can quantify sustainability metrics by aggregating information from the process level, where various metrics require different aggregation methods, from the manufacturing process to the manufacturing system level.

The work of Garretson et al. [22] facilitated standards development efforts by harmonizing the terms used to describe production processes. A set of 47 terms focusing on process characterization and describing sustainable production was generated, although terms unique to individual production processes were omitted. The terms were organized into six categories to define the overall concepts: Scope, Boundary, Material, Measurement, Model, and Flow. Then, definitions of the terms were derived from: (a) the literature in sustainable manufacturing and chemical and process industries, (b) process characterization and planning, (c) organization standards, and life cycle assessment and management. The reported terms and definitions are not unique to sustainable production.

Helleno et al. [23] proposed a conceptual method of integrating a new group of sustainability indicators into the Value Stream Mapping (VSM) tool to assess manufacturing processes. The method was applied in three case studies, and the results demonstrated that the proposed method identified different levels of manufacturing process sustainability and thus enabled the development of improved scenarios. 

Kluczek [24] introduced an “improvement scenario” in a company producing heating devices, between existing and new processes, based on an approach that can be applied to perform the sustainability assessment of manufacturing processes, requiring less detailed data, time, and expert knowledge but still providing a company-level analysis.

Latif et al. [25] developed an interactive model to determine the sustainability index based on user responses; the model is able to provide suggestions to improve sustainability, as well as carbon footprint reduction, and can assist industry to identify its shortcomings in achieving sustainability, can determine the carbon footprint reduction potential, and can compare the sustainability index as a benchmark measure.

Moldavska and Welo [26] analyzed the different definitions of sustainable manufacturing (SM) and identified the current understanding using an inductive content analysis of definitions published in a variety of academic journals. It is proposed that the findings can serve as a foundation for the development of a common language in both the research field and industrial practice.

In the literature reviewed, Winroth et al. [27] compiled the existing Sustainable Framework of Indicators, in which sustainability assessments can be carried out at different levels within an organization.

**Figure 1:**
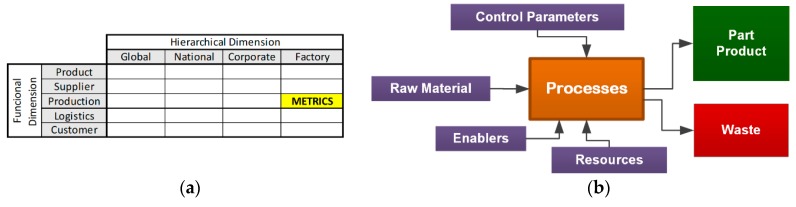
Dimensions selection sustainability indicators modeling. (**a**) Criteria for Metrics Selection [27]. (**b**) Generic Process Flow Diagram [28].

The hierarchical dimension of the activities (global, national, corporate, or factory), and the functional dimension (product, supplier, production, logistics, and customer) are shown in Figure 1a. This analysis allows us to select the production indicators at the factory level, at which in each industrial process there are proposals for the measurement of sustainability, among which manufacturing companies use the key performance indicators (KPIs) for the control and monitoring of their processes for continuous improvement.

For example, Linke et al. [28] proposed a generic process diagram: resources (raw material, energy, auxiliary materials, etc.) and enablers (the machine, the worker, the tools, etc.) are considered as inputs to the process, as shown in Figure 1b. 

The process can be evaluated through performance parameters, indicators, or metrics, which can be related not only to outputs but also to resources, enablers, and inputs in order to give feedback during the manufacturing phase of the product life cycle. The final quality of the manufactured part or the assembled product will be achieved by controlling activities but also by obtaining data and information from the metrics. The waste output generated from the process will be in the form of physical material or environmental pollution.

From the perspective of manufacturing throughout the product life cycle, industrial processes can be analyzed by considering stages such as the extraction of raw materials, manufactured materials, product manufacturing, shipping, distribution, use, recycling, and the final disposal of the product. It is important to emphasize that a certain percentage of the recycled material can be integrated back into the material manufacturing process, while the rest must be used in other applications. 

Vila et al. [29] proposed a framework for defining a structured set of metrics that are customizable for operations in different manufacturing technologies. Although the research work was applied to AISI 1018 material turning operations in order to analyze the surface integrity of the part, the contribution established the relationships between the machining parameters of the turning process and the final properties of the manufactured part, such as roughness, microhardness, and other parameters. This was the first attempt to link general sustainable metrics with technological-related metrics. For this reason, several perspectives were proposed. Table 1 shows Product, Process, and Resources (PPR) perspectives and some activity indicators that can be defined during the product life cycle. High-level general indicators aligned with sustainable objectives are initially created for each activity.

With this general view of manufacturing sustainable metrics, the next step was to explore previous works in order to define a sustainable scorecard indicator for machining or material removal techniques.

### 2.2. Dimensions of the Sustainability Metrics for Machining Processes

In the review of the state of the art, we can find some contributions that define sustainable metrics and show how important it is to define them at different levels of the product life cycle. One of the most interesting works was done by Bhanot et al. [30], in which they analyzed the complex interdependences between parameters that affect the result of a metal cutting process when seeking sustainable objectives. From the literature and from other previous works, we can highlight the manufacturing aspects to measure. 

In manufacturing, it is critical to guarantee the competitiveness of each activity from many points of view, and a balanced optimization between the economic, environmental, and social dimensions must be obtained. Therefore, it is necessary to define not only the technological metrics, but also the sustainability metrics aligned with the manufacturing process. For example, for each dimension, we can list some aggregated metrics:

(a) Economic Dimension: Surface Roughness, Material Removal Rate (MRR), Tool Life per Edge, Production Rate per Edge, Production Cost per Component, Process and Production Management.
(b) Environmental Dimension: Coolant Consumption, Carbon Emission, Energy Consumption, Cutting Temperature, Recyclable Waste Production, Non-Recyclable Waste Production, Waste Management.
(c) Social Dimension: Individual Productivity, Relations with Other Workers, Worker Skills, Rotation Flexibility at Work, Punctuality at Work, Senior Management Support, Total Satisfaction, Suspicious Work Environment, Degree of Support from Authorities, Compliance with Worker Requirements.

The indicators in the economic and environmental dimensions can be defined through analytic expressions, and they can be quantitatively evaluated using data mining and process calculations. In the social dimension, the indicators are mainly evaluated qualitatively. Some of these indicators are introduced in Table 2. According to the product life cycle phase and for different PPR perspectives—Process, for example—we can find technical metrics associated with the economic dimension (Material Removal Rate), the environment dimension (Cutting Temperature), or the social dimension (Worker Skills).

The appropriate selection of sustainable indicators allows for the diagnosis of continuous improvement plans in industrial processes, especially in manufacturing processes. However, it is still difficult to define social metrics in manufacturing activities, as Ayabaca and Vila presented in their work and, moreover, to analyze the data [31].

Bhanot et al. [32] presented a study on a machining group in which the interdependencies of different sustainable machining parameters were examined in the context of milling and turning processes. 

In order to ensure competitiveness in the manufacturing field, there must be a balance between the economic, environmental, and social dimensions. Gupta et al. [33] presented an experimental investigation that compared empirical and experimental results, which was complemented by a desirability optimization technique, to study the impact on cutting forces, surface roughness, tool wear, surface topography, microhardness, and surface chemical composition in turning the aerospace material titanium (grade-2) alloy, considering Minimum Quantity Lubrication (MQL) conditions. 

Hegab et al. [34] developed and discussed a sustainability assessment algorithm for machining processes. The four life cycle stages (pre-manufacturing, manufacturing, use, and post-use) are included in the proposed algorithm. Energy consumption, machining costs, waste management, environmental impact, and personal health and safety are used to express the overall sustainability assessment index. Kadam et al. [35] analyzed the surface integrity in high-speed machining of Inconel 718, and the results show that a good surface finish and residual stresses in compressive regimes can be ensured in the high-speed machining range with low MRR in a water-vapor machining environment, this also being feasible at high MRR in dry cutting.

Benedicto et al. [36] presented a comprehensive analysis of the use of cutting fluids and their main alternatives in machining, focusing on the economic, environmental, and technical dimensions. Zhao et al. [37] reviewed a critical assessment of energy consumption in a machining system at the process, machine, and system levels. Machine tool power demands in different machine states with different components were also discussed, and the predictive methods of energy consumption at different levels were summarized. Energy consumption reduction strategies to achieve sustainable manufacturing were also discussed.

Abbas et al. [38] presented an extensive study of the effectiveness of using different cooling and lubrication techniques when turning AISI 1045 steel. Three multi-objective optimization models were employed to select the optimal cutting conditions. The results offer a clear guideline for selecting the optimal cutting conditions based on different scenarios: MQL nanofluid compared to dry and flood approaches.

Ali et al. [39] found that the tool path strategy has a significant influence on the end outcomes of face milling, considering the surface topography with respect to different cutter path strategies and the optimal cutting strategy for the material Al 2024. Li et al. [40] evaluated the cutting performance of cutting tools in the high-speed machining (HSM) of AISI 4340 by using tools coated with TiN/TiCN/TiAlN multi-coating, TiAlN + TiN coating, TiCN + NbC coating, and AlTiN coating, respectively. A TiN/TiCN/TiAlN multi-coated tool is the most suitable for the high-speed milling of AISI 4340 due to the lower cutting force, lower cutting temperature, and high diffusion resistance of the material. Gupta et al. [41] discussed the features of two innovative techniques for machining an Inconel-800 superalloy by plain turning while considering some critical parameters, reducing the amount of cutting fluid while using sustainable methods. Near dry machining (NDM) will be possible and will solve the problem of chemical components in the fluids being harmful to human health.

In recent research, Gamage et al. [42] used a Taguchi design of experiments and analysis of variance (ANOVA) to identify the significant parameters that optimize the process energy consumption of wire electro-discharge machining (WEDM) of the superalloys Inconel-718 and Ti64Al4V. The results indicate that the preferred parameters to minimize the specific energy consumption are workpiece thickness, wire material, wire diameter, and pulse-OFF time. The reduction of carbon emissions corresponds to the non-working energy consumption of the machines, which is also calculated.

Gunda et al. [43] presented a novel technique for the generation of machining techniques—namely, high-pressure minimum quantity solid lubricant (HP-MQSL) and an experimental setup, with an aim of improving process performance and eliminating the use of cutting fluids in machining operations.

Lu and Jawahir [44] presented a sustainability evaluation methodology for manufacturing processes based on cryogenic machining processes which involves a metrics-based Process Sustainability Index (ProcSI) evaluation. This helps to decide the best cutting conditions from the sustainable manufacturing viewpoint.

Pusavec et al. [45] presented an experimental study of the sustainable high-performance machining of Inconel 718 with the development of performance-based predictive models for dry, near-dry (MQL), cryogenic, and cryolubrication (cryogenic þ near-dry) machining processes using the response surface methodology (RSM). The models developed in the first part of the paper are used in the second part for process evaluation and optimization, to determine the optimum machining conditions for an overall process performance improvement. 

Goindi and Sarkar [46] presented a review of all aspects of dry machining, including the sustainability aspects of machining, especially focusing on three research objectives: (1) identifying the areas where dry machining has been successfully adopted and where it has not been possible to do so, (2) reporting on the research work carried out and various alternative solutions provided by the researchers in the area of dry machining, and (3) finding gaps in the current knowledge and suggesting some directions for further work to make dry machining more sustainable, profitable, and adaptable to product manufacturing. Shin et al. [47] presented a component-based energy-modeling methodology to implement the online optimization needed for real-time control in a milling machine. Models that can predict energy up to the tool path level at specific machining configurations are called component models.

Um et al. [48] proposed an approach for deriving an energy estimation model from general key performance indicators of the sustainability of machine tools in the laser welding process of an automotive assembly line and the milling process of an aircraft part manufacturer. ANOVA and RSM are widely used for optimizing cutting parameter tools. Zhang et al. [49] proposed using the Pareto diagram to calculate multi-objective optimization, although this is difficult when there are more than two objectives. This proposal lists and characterizes all the 128 scenarios of sustainable machining operations, considering seven objectives that include energy, cost, time, power, shear force, tool life, and surface finish. The results show that all the scenarios can be converted into a simple objective situation that has a single solution or a set of contradictory bi-objective cases that can be represented on a simple Pareto front.

The use and storage of the calculated indicators, together with modern systems of data acquisition and information management in real time, will strengthen the implementation of advanced manufacturing systems, or what is called Industry 4.0. Activities such as team maintenance and specific service requirements can be planned and adjusted in real time. Gao et al. [50] reviewed the historical development of prognosis theories and techniques and projected their future growth in the emerging cloud infrastructure.

### 2.3. Sustainable Metrics for Manufacturing Processes

**Figure 2:**
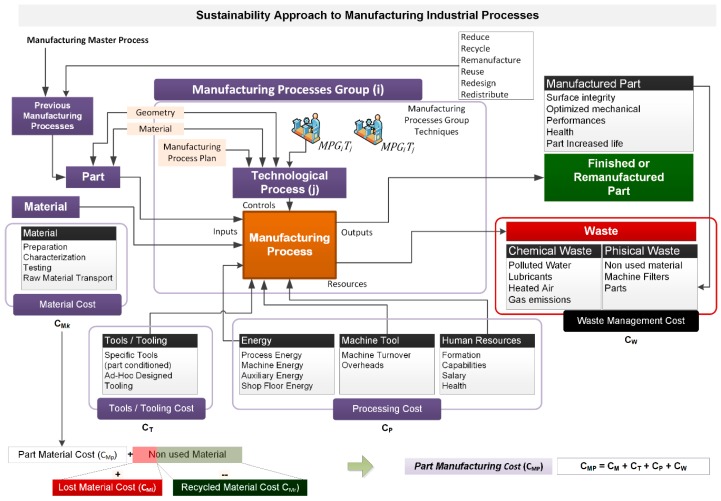
Activity model of a generic industrial manufacturing processes.

The first contribution of the research after the review of the state of the art is shown in Figure 2, which gives the Sustainability Approach to Manufacturing Industrial Processes and summarizes the stages of the product life cycle: inputs, enablers, manufactured parts, and waste. This framework was outlined after a deep analysis of manufacturing industrial processes and activities. The activities model was sketched using ICAM Definition (IDEF) methodologies [51] and Unified Modeling Language (UML). Figure 2 presents the top level from the hierarchical model that is later deployed in different layers with more detail of activities and customized for different manufacturing technologies. 

The activity model includes, for a manufacturing process, the inputs, which can be either raw material or a geometrical preform from previous manufacturing technology. The input of controls depends on the manufacturing technique, and it is represented here as a Manufacturing Process Group Technique (MPGT). For each manufacturing technology group (i), we can find different variations or techniques (j). The evaluation of the technological process parameters, according to the manufacturing process plan (control), will assure the quality of the final product or the quality of an individual part. 

The controls parameters will depend on the technological process that is applied, while the performance parameters, which can be also introduced, define the process efficiency.

The model describes the general enablers and resources that are grouped into tools and tooling, energy consumption, machine tools, and human resources. For each enabler and resource, we can analyze the different issues and therefore define specific metrics. For example, the model shows countable metrics such as the machine-consumed energy or uncountable metrics such as works formation.

Finally, an important issue is to define the economic impact considering all the activities, inputs, controls, resources, enablers, and outputs. The costs of part manufacturing can be calculated considering the material costs (C_M_), the cost of tools (C_T_), the costs of the process (C_P_), and the costs of waste management (C_W_).

For the final output, the analysis of all the metrics defined for inputs, controls, enablers, and resources, for each product life cycle phase and from different perspectives, will help to establish whether the result meets the technical, functional, and sustainability requirements.

With this activity model, the next step is to validate it for a manufacturing process and technology that, in our research, will be material removal or machining processes and technologies.

### 2.4. Sustainability Metrics for Machining Operations

At the factory level, a manufacturing technology or technique requires one to define correctly the process plan so that the processes, equipment, people, etc., in the production system perform a specific function. Manufacturing process engineering requires a clear and complete description of the information associated with the process and the exchange of data to be of such a quality that forecasts of the results can be obtained. The modeling of systems and processes is expected to standardize the process—what is done, what is controlled, what resources are required—and define the products generated.

For the improvement of industrial quality, a knowledge of industrial processes is required. In this case, we will analyze the process in which it is defined: function, inputs, outputs, resources, and controls that allow measuring performance, as well as the emissions generated, which are evaluated in the context of sustainability.

**Figure 3:**
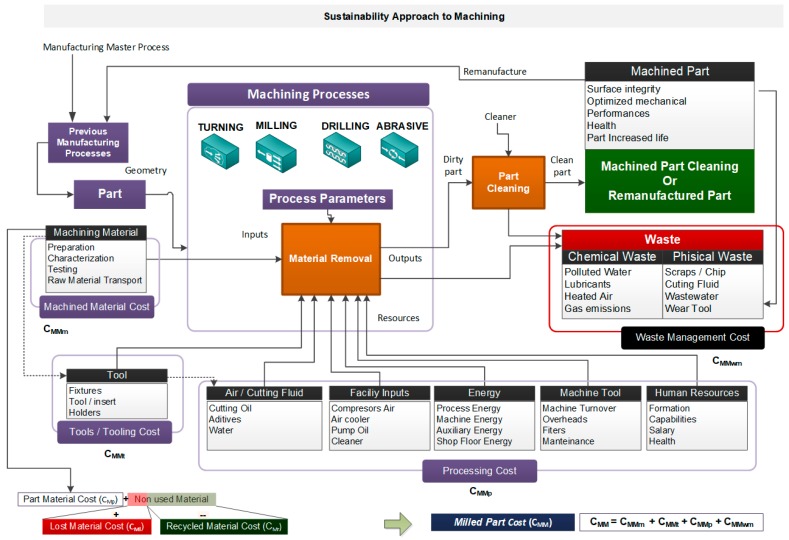
Activity model for machining operations and metrics definition.

The second contribution of this research is the matching of the previous general model in a specific model for machining processes and technologies and the definition of metrics in each phase for different machining activities and detailed operations. The activity model for machining is shown in Figure 3, and it represents the material inputs, cutting tool preparation, and the different resources used for machining: cutting fluids, compressed air, energy consumption, facilities consumption, machine tool use, and repayment and human resources.

For machining processes and technologies, the activity model was defined considering the following basic issues:

- MAIN ACTIVITY: material removal or machining. The most common machining processes and technologies in industrial shop floors are turning, milling, drilling, and grinding, although this activity model can be similar to advanced machining processes and technologies such as chemical machining, electrochemical machining, thermal machining (laser cutting), or advanced mechanical machining (water cutting).
- INPUTS: materials to obtain the final part; in this case, the preform to be machined. In this manufacturing process, we consider the material characterization, testing, preparation, and transport as inputs.
- RESOURCES: cutting tools, compressed air, cutting fluids, facility inputs, energy, machine tools, and human resources. These resources are different depending on the individual operation defined in the macro and micro manufacturing process plan. For example, regarding the consumed energy, we will define the metrics for machine tool consumed energy and cutting fluids consumed energy, as well as the compressed air requirement for the operation and other auxiliary systems.
- CONTROLS: technological instructions and process indicators defined in the micro manufacturing process plan, which ensure the efficiency and effectiveness of the process in order to obtain the final product. Apart from these, we introduce indicators that can be evaluated from the sustainability perspective, considering economic, environmental, and social dimensions.
- OUTPUTS: The final machined part must be cleaned at the end of the process, since it generally uses cutting fluids with chemical agents. However, the most important issue is that the process generates removed material in chips that we must manage and recycle.
- WASTE: Although it is desired to minimize the total waste, depending on the number of different machining phases, we can divide this metric for each one. We consider scrap or residuum generated by the production process—this can be physical (chips, raw material details, or broken cutting tools), chemical (used cutting fluids mixed with microscopic chips or wastewater) or air pollution, due to gas emissions.

**Figure 4:**
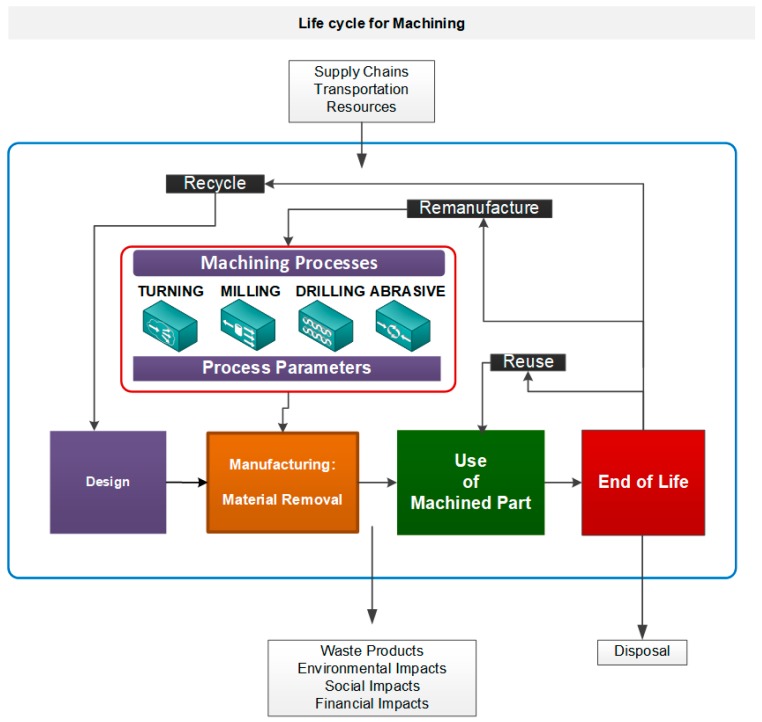
Main product life cycle phases where the activity model is positioned.

Nevertheless, this activity model is not enough if we want to design a balanced scorecard that includes sustainability indicators. It is obvious that a product’s design will have a great influence on how it is manufactured and what materials, processes, and systems are used, as one of the specialists in green manufacturing, Dornfeld [52], indicated. These contributions to sustainability indicators provide the basis for acquiring data during the product life cycle. We propose four main phases for the product life cycle, which include design, manufacturing, use, and end of life, as shown in Figure 4, in order to locate the proposed activity model.

The approach to these four phases will allow us to define indicators in each one from the PPR perspective

1. Design. This phase includes raw material management and product design and development stages. To design indicators, we consider not only materials flowing from mining but also from recycled products and cause–effect actions on next phases in engineering activities.
2. Manufacturing. In our research, we consider material removal, machining, processes, and technologies, and the proposed activity model (Figure 3) is incorporated into this phase and we will mainly present indicators here.
3. Use. This third phase is related to product use and service. Thus, we will focus on individual part maintenance or spare parts.
4. End of life. This phase supposes the disposal or the recycling of products once obsolescence is reached and, therefore, individual part disposal or recycling according to companies’ sustainability strategy.

Supply chains and materials transport are sometimes considered a separate life cycle phase, but from our point of view, transport and distribution happen throughout the product’s life cycle. The complete supply chain is also an integral part of the product life cycle, as these supply chains must produce, deliver, and collect a finished good for use or at the end of its life.

With the activity model for machining and the product life cycle framework, we define the sustainability metrics in our research.

## 3. A Framework for Sustainability Machining Metrics

**Figure 5:**
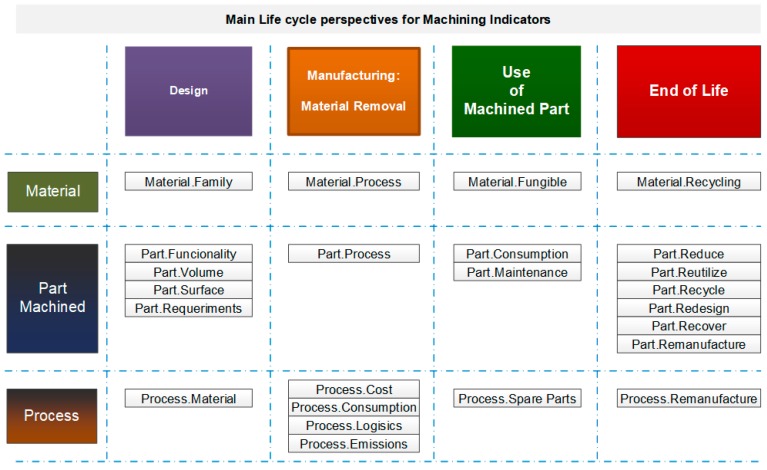
Machining indicators from different PPR perspectives along the product life cycle.

The proposal includes the definition of grouped indicators from the PPR perspective. In this work, we will not include resource indicators, and the Product perspective will be subdivided into Materials and Parts. The name of the grouped indicator will start with the PPR name (Material, Part, or Process) followed by another name related to what we want to measure, as shown in Figure 5.

In order to describe the indicators, we have divided the proposal into phases. Table 3 shows how the specific information is organized in this section, and the following tables contain detailed information about the indicators per phase and per PPR perspective.

The definition of the indicators can be used to create a balanced scorecard aligned with a company’s sustainability strategy. The first approach to specific indicators is shown in Table 4, Table 5, Table 6, Table 7 and Table 8, and they include both quantitative and qualitative indicators that can be used all along the product life cycle and its name and a short description.

For the first product life cycle phase, Design, the design considerations are strengthened by the search for new materials that have high performances for parts and have allowed a wide range of manufacturing options. High-performance materials and new mechanical and chemical characteristics are incorporated into the databases in product life cycle management (PLM) platforms and help engineers to make the right decision. Table 4 shows the general definitions for the Design phase.

In machining processes, the relationship between the material, geometry, cutting tool, and machine tool opens the field to the research in manufacturing process optimization and new materials. The operating conditions (process parameters for machining) depend on the efficient performance of these four elements. Table 5 describes, for the manufacturing phase, the indicators for the machining process, including turning, milling, drilling, and boring operations, among others.

Machine tools can be manual, automated, or numerical control-driven. Today, most of them are ready for Industry 4.0 connection, and CAD/CAE/CAM applications are associated with shop floor cells and provide data to PLM platforms. The manufacturer’s recommended tool parameters (controls) are based on extensive studies of the process, part material, part geometry, and tool performances. With appropriate sustainable indicators, we can improve them. For example, the energy consumption of the process and its emissions metrics will require experimental measurements on the shop floor to establish the process indicators and online data collection.

Machined parts used as machine components or consumer product components must meet the design specifications, in which the consumables and the consumption of energy sources for their operation must be considered. Table 6 shows the definitions of metrics from this perspective.

The end of life of a machined part can be postponed by maintenance and repairs, which may include processes for the recovery of dimensional tolerances, for which an analysis of surface integrity may be necessary. Table 7 shows the main definitions.

The specific information regarding some indicators is shown in Table 8. The table shows the indicator, the simplified name, acronym, units, goal, and the possible source of the information. The indicator units depend on the specific variables that are measured, and the objective may be seeking the maximum (↗) or the minimum (↘). The sources of information can be standards or databases of materials, machines, tools, and consumables, which can be taken as a reference for the analyzed process.

## 4. Experimental Development, Results, and Discussion

In order to validate the metrics definition, a set of experiments were designed. The experiments had the objective of acquiring data for some indicators and to analyze how variations on manufacturing process plan parameters could affect the sustainable indicators.

The experiments were included in a green product life cycle management initiative and, for example, advanced Computer Aided Design and Manufacturing (CAD/CAM) tools were used to prepare the design of experiments for different indicators. It should also be noted that the proposal managed high-level and low-level indicators and indicators in different phases that can be validated on the shop floor. For example, machining time can be determined through the design phase indicators with CAM applications, and the real operation time can be measured in the machine tool numerical control and then compared.

The experiments are summarized in Table 9 and, for each one, the indicators from the PPR perspective are evaluated. For each experiment, a basic machining technology was tested with previous machining simulation arrangements.

In the following subsections, the five experiments are briefly described to show how to obtain data for the defined sustainable indicators.

### 4.1. Test #1. Material Removal Rate (MRR) and Machining Time

The objective of this experiment was to set the minimum processing time and the highest possible MRR in a planning process. The design of the experiments considered cutting directions of 0°, 45°, and 90°, and the material was AISI1045 in the Gentiger machining tool (Taichung City, Taiwan), with the cutting tool Mitsubishi VPX300R 4004SA32SA and LOGU1207080PNER-M (MP6120) inserts (Tsukuba, Japan). The cutting diameter Ø = 40 mm, the number of flutes Zc = 4, and the main cutting angle K = 90°. The operation was done in all cases without cutting fluids.

The CAD/CAM Inventor HSM 2019 application was used to find the 27 possible combinations of the Taguchi method to get the combination of four parameters (ABCD) that reaches the highest MRR and the minimum processing time. In this experiment, A is the direction of the cutting trajectory pass *p_d_*, B is the depth of cut *a_p_*, C is the cutting speed *v_c_*, and D is the feed rate per tooth *f_z_*. The time is expressed in min:s.

The indicator, shown in Table 9, revealed that the highest MRR material removal rate obtained for the roughing operations was MRR = 10.1 cm^3^/min. In this operation, the control parameters were *p_d_* = (0° or 45° or 90°), *a_p_* = 1.2, *v_c_* = 1671, and *f_z_* = (0.10 or 0.12 or 0.08). 

For the finishing machining operation, the highest material removal rate obtained was MRR = 6.1 cm^3^/min. In this operation, the control parameters were *p_d_* = (0° or 45° or 90°); *a_p_* = 0.8; *v_c_* = 1671; and *f_z_* = (0.12 or 0.08 or 0.10).

Finally, the minimum machining time obtained for the finishing operation was *t_min_* = 2:25 min:s. The process parameters were *p_d_* = 0°, *a_p_* = 0.8, *v_c_* = 1671, and *f_z_* = 0.12.

### 4.2. Test #2 Machining Strategies on Concave and Convex Surfaces

The objective of this experiment was to find the machining strategy with the shortest machining time among the possible options of the cutting trajectories strategy. The surface geometry was specially designed for the test, and the machined material was AISI1045. 

The experimental setup used a high-performance Gentiger machining center, and the cutting tools for surface machining were the Mitsubishi Ball Mill VQ4SVBR0600 (cutting diameter Ø 6 mm) for roughing surface milling and the Mitsubishi VQ4SVBR0300 (cutting diameter Ø 3 mm) for the finishing operations from Tsukuba production centre (Japan). Both were done with the recommended cutting fluids, which encouraged us to optimize its use for sustainability reasons. All the experiments were prepared with the CAM application of the 3DEXPERIENCE 2019 platform. In this case, 21 different options were achieved.

Several evaluations were made on the part that had a concave and convex surface. The test analyzed different options for tool path generation and trajectory strategies in three-dimensional surface milling with all the possible combinations and simulations [53]. For example, with the *style pocketing* surface machining option and the *back and forth* strategy, it was found that the total operation time was reduced by 10%, while only a 3% time reduction could be achieved for the rest of the options compared to the longest one.

In this experiment, we could obtain qualitative information about the cutting trajectory strategy and quantitative information about the operation time and, therefore, the energy consumption. 

### 4.3. Test #3. Roughness, Microhardness, Plastic Deformation

The objective of this experiment was to find the effect of the machining parameters on the surface quality of the part, which was a quality requirement. The part was a machine axis, and the main machining operations were turning. The part material was AISI1018, and the machine tool was the ROMI numerical control lathe (Sao Paulo, Brasil).

The turning operation cutting tool used was the SANDVIK DNMG 15 06 08-PM4325, and the operations were done with cutting fluids. The machining microprocess plan with trajectory strategies was prepared with the CAD/CAM application SolidCAM 2018 [29].

Mathematical relationships were found to predict these properties and recommendations for the use of different parameters. This test used experimental data from a turning process to determine the influence of the machining parameters in the surface quality, such as the depth of cut, yield strength, plastic deformation, and roughness. The analytical indicator helped us to decide which combination of parameters can be accepted or rejected according to the requirements and dimensional tolerances of the part. Experimental equations were obtained for the selected process, machine, material, and tool.

### 4.4. Test #4: Roughness and Power Consumption

The objective of this experiment was to reproduce the machining process plan to build the same part in two different machining centers (A and B) that were geographically distributed. The experiment was reproduced with exactly the same machining process parameters, and the aim was to determine whether the minimum roughness and the minimum power consumption would be obtained with the same parameters [54].

The experiment was carried out in a high-performance Gentiger Machining Center (Taichung City, Taiwan) (A) and in a high performance Deckel Maho Machining Center (Pfronten, Germany) (B).

The part material was AISI1045, and the cutting tool was the Mitsubishi VPX300R 4004SA32SA, with LOGU1207080PNER-M inserts (MP6120). The cutting diameter Ø = 40 mm, the number of flutes Zc = 4, and the main cutting angle K = 90°. The operation was dry machining, without cutting fluids.

Although both experiments had the same machining cutting parameters, it was discovered that the machine has an important influence on the result and, therefore, on the micro process plan.

**Power consumption**. In Machine #A, the minimum power consumption was 2.79 kWh, with the conditions of a pass direction of 90°, a cutting depth of 1.0 mm, a cutting speed of 180 m/min, and a feed per tooth of 0.1 mm/tooth. In Machine #B, the minimum power consumption was 4.88 kWh, with the cutting conditions being a pass direction of 0°, a cutting depth of 0.8 mm, a cutting speed of 140 m/min, and a feed per tooth of 0.08 mm/tooth.

**Roughness**. In Machine #A, the minimum value of R_a_ = 0.55 μm, with the cutting conditions of a pass direction of 0°, a cutting depth of 0.8 mm, a cutting speed of 210 m/min, and a feed per tooth of 0.12 mm/tooth. In Machine #B, the minimum value of R_a_ = 0.83 μm, with the cutting conditions of a pass direction of 45 °, a cutting depth of 0.8 mm, a cutting speed of 210 m/min, and a feed per tooth of 0.08 mm/tooth.

The conclusion was that although we had twin machine tools with similar performances, we have to slightly customize the process plan to reach the indicator objective.

### 4.5. Test #5. Social Dimension Analysis

For this experiment, the evaluation of the social dimension in machining was the main objective. The experiment was carried out on a shop floor with numerical control machine tools that perform machining processes on various parts at the same time.

An assessment questionnaire was designed and completed by workers, shop floor officers, and middle managers, obtaining the minimum number of answers to validate the method.

The indicators were calculated by the grey relational theory. There were 16 indexes analyzed: Worker Productivity, Relations with Other Workers, Worker’s Skill Level, Flexibility of Job Rotation, Punctuality, Top Management Support on Various Issues, Job Satisfaction, Conducive Working Environment, Awareness of Sustainable Manufacturing Initiatives, Technological Upgrades, Financial Support (loans, etc.), Required Product Quality, and Waste Management [31].

The obtained results when evaluating the sustainability indicators in the social dimension, after the application of the Plan, Do, Check, Act (PDCA) continuous improvement cycle were 9.21% higher than the initial evaluation after the implementation of the improvements.

Some improvements were implemented after the analysis of the initial evaluation and the final evaluation of this sustainability dimension, which is one of the most difficult to get information and data analysis for. Fortunately, it helped to implement objective indicators on the machining shop floor. 

## 5. Conclusions

This paper’s contributions can be highlighted in three main areas of interest that have been presented, from the indicators’ definition to the shop floor in machining operations. 

The first one is the general activity model of industrial manufacturing processes that can be deployed in more detailed activities to identify indicators, where needed, for the manufacturing phase of the product life cycle management.

The second one is the customization of the activity model for material removal and machining processes. In this model, we detected the manufacturing machining process inputs, controls, resources, and enablers in order to define the general, technical, and sustainability indicators. These indicators can be defined for different product life cycle phases in an organized way, which is why we defined the PPR life cycle phases matrix.

The different experiments carried out provided skills, data, and information about the applied indicators in the manufacturing and materials engineering discipline. They gave real case studies for validating the metrics’ definition.

Apart from the manufacturing phase metrics’ definition, the use of manufacturing authoring applications within a PLM platform in the design stage can simulate the manufacturing process and help to predict its behavior under the required conditions. Computer-aided manufacturing simulation software has different simulation levels that can help to define and validate machining strategies or manufacturing cell activities to ensure good product quality, achieving sustainable strategies.

In the comparative study of the two machining centers, the lowest roughness and the lowest energy consumption were obtained with different machining parameters. The experiment was carried out on the same material and the same tool, and it was determined that sustainability indicators must be established for each machining center.

When considering surface roughness and the power consumed as the variables to find the best cutting conditions, it was determined that each machining center has its own operating parameters for these conditions. These parameters are related to each other, and the value depends on the material selected, the tool, the machine center, and the lubrication.

The manufacturing parameters that can be tested with virtual manufacturing help to minimize the iterative process when fixing the indicators’ objective values. In other cases, it will be more difficult, and we will need to do shop floor measures, as shown in the last experiment.

Finally, sustainability indicators should be evaluated in the product design stage for the best results, and the characteristics of the manufactured part can best be predicted by including the sustainability criteria in the product life cycle management (PLM) platforms.

## 6. Future Work

The research plans aligned with this proposal, and we learned multiple lessons, including several key actions. Firstly, we suggest a proposal to incorporate indicators’ reports into product life cycle management platforms, in which the sustainability alternatives proposed in the design stage can be evaluated, as part of digital twins’ implementation for Industry 4.0 demonstrators. Secondly, further research should carry out experiments to determine the influence of mooring in milling processes and its influence on the quality of the part and the sustainability indicators. Finally, future research should focus on developing a system for evaluating sustainability indicators that can quantify the increase in the indicators when the product is improved, while the options or alternatives are analyzed in the design and manufacturing stages.

## Figures and Tables

**Table 1:** Product, material, and resources activities as general performance indicators.

PPR Perspective	Activity	Generic Indicator	Units	Sustainable Objective
Material	Raw Materials Extraction	Material class Material properties	Options Value	↗ ↗
	Manufacture	% Recycled content Weight	% Kg	↗ ↘
Product	Transport and Distribution	Region where it is manufactured. Transportation to end user.	Km, CO_2_ M	↘ ↘
	Use	Energy need throughout its useful life	kWh	↘
	Recycling	% Recycling	%	↗
	End Disposition	% Burned % Spell	% %	↘ ↘
Process	Product Manufacture	Energy of the manufacturing process. Useful product lifetime of the product. Energy needed for assembly.	kWh s kWh	↘ ↗ ↘

Note: Objectives symbol’s meaning ↗ maximize; ↘ minimize.

**Table 2:** Generic indicators for sustainable machining. PPR: Product, Process, and Resources.

PPR Perspective	Phase	Generic Indicator	Acronyms	Units	Sustainable Dimension
Product	Use End Disposition	Surface Roughness	R_a_	µ	Economic
		Refrigerant Consumption	R_c_	m³	Environmental
		Carbon Emissions	Ce	CO_2_	Environmental
Process	Product Manufacture	Material Remove Rate	MRR	m^3^/s	Economic
		Tool Life per Edge	T.L/edge	Min	Economic
		Production Rate per Edge	PR/edge	Units	Economic
		Production Cost per Component	PC/edge	€/part	Economic
		Energy Consumption	Ec	kWh	Environmental
		Cutting Temperature	Ct	°	Environmental
		Worker Productivity	Wp	%	Social
		Relations with Other Workers	Rw	%	Social
		Worker Skills	Ws	%	Social
		Rotation Flexibility at Work	Rf	%	Social
		Punctuality at Work	Pw	%	Social
		Senior Management Support	Sms	%	Social
		Total Satisfaction	Ts	%	Social
		Auspicious Work Environment	Awe	%	Social
		Support from Authorities	Sfa	%	Social
		Worker Requirements	Wr	%	Social

**Table 3:** General and specific information on sustainable machining indicators.

Phase	General Information	Detailed Information
Design	Definitions of Material, Product, and Process in the stage of design (Table 4)	In Table 8, specific information and definitions of Material, Product, and Process in the design stage, manufacturing, use and end of life.
Manufacturing	Definitions of Material, Product, and Process in the stage of manufacturing (Table 5)	
Use	Definitions of Material, Product, and Process in the use stage (Table 6)	
End of Life	Definitions of Material, Product, and Process in the end-of-life Stage (Table 7)	

**Table 4:** Design phase indicators definition.

Phase	PPR Perspective	Indicator Name	Description
Design	Material	Material.Family	Identifies the materials used for the design of each component of the product
	Product	Part.Functionality	Describes the function of the part and determines the mechanical relationships with the others in the set
		Part.Volume	Indicates the volume of a part. The mathematical definition depends on its geometry
		Part.Surface	Reveals the surface integrity of a part/product
		Part.Requirements	Lists the functional requirements of the part, for example roughness, dimensional tolerances, etc.
	Process	Process.Material	Illustrates design considerations that affect compatibility between manufacturing processes and the selected material group

**Table 5:** Manufacturing phase indicators definition.

Phase	PPR Perspective	Indicator Name	Description
Manufacturing	Material	Material.Process	Establishes the design considerations that affect the compatibility between the material selected with the manufacturing process
	Product	Part.Process	Illustrates the design considerations that affect the geometric compatibility of the part with the manufacturing process.
	Process	Process.Cost	Reveals the cost of the process per part/unit
		Process.Consumption	Shows the energy consumption in the manufacture of the part. This indicator contains more detailed indicators for each energy source (W, L/h, etc.)
		Process.Logistics	Describes the material flow, internal and external to the shop floor. This indicator contains more detailed indicators according to the part process plan
		Process.Emissions	Indicates the emissions of solids, liquids, and gases produced in the process. This indicator contains lower-level indicators with different perspectives.

**Table 6:** Use phase indicators definition.

Phase	PPR Perspective	Indicator Name	Description
Use	Material	Material.Fungible	Indicates the necessary materials or components used by the product during its phase of use
	Product	Part.Consumption	Indicates the consumption of various energy sources used by the product for proper operation (water, electricity, gas, etc.)
		Part.Maintenance	Lists the maintenance actions that must be undertaken during use, mainly those programmed, with an estimate of unscheduled maintenance
	Process	Process.SpareParts	Shows the manufacturing orders that must be issued to maintain the legally established stock of the product during the use phase and after production ends

**Table 7:** End-of-life phase indicators definition.

Phase	PPR Perspective	Indicator Name	Description
End of Life	Material	Material.Recycling	Identifies the amount of material that can be recycled for each of the parts/components used in the product
	Product	Part.Reduce	Identifies the parts or components that can be removed without damaging the proper functioning of the product
		Part.Reutilize	Identifies the parts or components that can be reused as components of another new product
		Part.Recycle	Identifies the parts or components that can be recycled and included as part of the base material as spare parts without damaging the proper functioning of the product
		Part.Redesign	Identifies parts or components that are likely to be redesigned to minimize the environmental impact of the assembly
		Part.Recover	Identifies the parts or components that can be recovered as spare parts without damaging the proper functioning of the product
		Part.Remanufacture	Identifies the parts or components that can be re-passed through a new manufacturing process and incorporated into a new product.
	Process	Process.Remanufacturing	Establishes the ability of the manufacturing process to form materials from a product in the end-of-life phase

**Table 8:** Specific information regarding the Indicators from the main life cycle perspective.

Phase	PPR Perspective	Indicator Definition	Expression	Units [Example]	Goal	Source of Information
Design	Material	Material.Family	M.F.	kg	↗	Standard/Databases
	Product	Part.Functionality	P.F	Functionality	↗	Guides
		Part.Volume	P.Vm	mm³	↗	@
		Part.Surface	P.Sf	µ; mm²	↗	@
		Part.Requirements	P.R	# Requirements	↗	@
	Process	Process.Material	P.Mc	Machining Operations	↘	Guides
Manufacturing	Material	Material.Process	M.Pc.	Machining strategy	↘	Standard/Guides
	Product	Part.Process	P.P	Machining path	↘	Guides
	Process	Process.Cost	P.C	€/unit	↘	@
		Process.Consumption	P.Co	Kg; €	↘	@
		Process.Logistics	P.L	s; m; €	↘	@
		Process.Emissions	P.E	Kg CO_2_	↘	@
Use	Material	Material.Fungible	M.Fu	Kg	↘	Database
	Product	Part.Consumption	P.C	kW/h	↘	Standards; @
		Part.Maintenance	P.M	OEE	↘	Guides; @
	Process	Process.Spare Parts	P.Rec	# orders	↘	Guides; @
End of Life	Material	Material.Recycling	M.R	Recycled Kg/Kg components	↗	Guides, Standard; @
	Product	Part.Reduce	P.Redu	parts/unit	↗	Guides; @
		Part.Reutilize	P.Reu	parts/product	↗	Guides; @
		Part.Recycle	P.Rec	parts/product	↗	Guides; @
		Part.Redesign	P.Reds	parts/product	↗	Guides; @
		Part.Recover	P.Rep	parts/product	↗	Guides; @
		Part.Remanufacture	P.Ref	parts/product	↗	Guides; @
	Process	Process. Remanufacturing	P.RMfg	%	↗	Guides; @

Note: ↗ maximize; ↘minimize; @ various information sources; # number of.

**Table 9:** Experimental validation of sustainable machining indicators. MRR: Material Removal Rate.

Phase	Test	PPR Perspective	Method	Machining Process	Material	Metric Evaluated	Goal
DESIGN	#1	Process	Simulation CAD/CAM Autodesk	Milling: Surface Facing	AISI1045	MRR	↗
						Machining Time	↘
	#2	Process	Simulator CAD/CAM 3DExperience	Milling: Concave Surfaces	AISI1045	Machining Strategies	↗
				Milling: Convex Surface	AISI1045	Machining Strategies	↗
MANUFACTURING	#3	Part	Measurements and tests	Turning Straight Turning	AISI1018	Roughness	↘
						Microhardness	↗
						Surface Metallographic	↗
						Mechanical Performance	↗
						Plastic Deformation	↘
	4#4	Part	Measurements between two machining centers	Milling: Surface Facing	AISI1045	Roughness (Machine Tool A)	↘
						Roughness (Machine Tool B)	↘
						Power Consumption (Machine Tool A)	↘
						Power Consumption (Machine Tool B)	↘
	#5	Process	Measurements in Social Dimension	Turning, Milling	Various	16 Sustainability Indicators	↗

Note: ↗ maximize; ↘ minimize.
